# Modelling dose and dose‐averaged linear energy transfer to predict high‐grade temporal lobe necrosis following skull‐base proton therapy

**DOI:** 10.1002/mp.70562

**Published:** 2026-07-25

**Authors:** Giulia Fontana, Elisa Fiorina, Sara Lillo, Lucia Pia Ciccone, Giulia Riva, Alessandro Vai, Mario Ciocca, Alberto Iannalfi, Silvia Molinelli, Ester Orlandi

**Affiliations:** ^1^ Clinical Department CNAO National Center for Oncological Hadrontherapy Pavia Italy; ^2^ Sezione di Torino Istituto Nazionale di Fisica Nucleare Turin Italy; ^3^ Department of Internal Medicine and Therapeutics University of Pavia Pavia Italy; ^4^ Radiation Oncology Unit, Clinical Department CNAO National Center for Oncological Hadrontherapy Pavia Italy; ^5^ Medical Physics Unit, Clinical Department CNAO National Center for Oncological Hadrontherapy Pavia Italy; ^6^ Department of Clinical‐Surgical, Diagnostic, and Pediatric Sciences University of Pavia Pavia Italy

**Keywords:** chondrosarcoma, dose averaged LET, proton therapy, skull‐base chordoma, temporal lobe necrosis

## Abstract

**Background:**

In skull‐base proton therapy (PT), severe toxicity outcomes such as temporal lobe necrosis (TLN) may be associated with inadequate management of the actual radiobiological effectiveness (RBE) of proton beams. Combining dose‐averaged linear energy transfer (LET_d_) and dose may be crucial in treatment plan optimization and evaluation.

**Purpose:**

To gather evidence on the combined effect of LET_d_ and dose on voxel‐ and structure‐wise levels in determining a high‐grade TLN (CTCAE v.5 grade ≥G2, G2‐TLN) in skull‐base tumors treated with PT; a dose‐LET_d_‐volume histogram (DLVH)‐based model was built to predict G2‐TLN, combined with an analysis of the Dose‐LET_d_ voxel‐based distributions.

**Methods:**

We retrospectively analyzed data of skull‐base chordomas (61) and chondrosarcomas (18), treated at CNAO National Center for Oncological Hadrontherapy (Pavia, Italy) with PT between September 2011 and July 2020, with a prescription dose of 74 and 70 Gy(RBE), respectively. RBE was set to a constant value of 1.1, while TL dose was constrained to D2cc < 71 Gy(RBE) in the optimization process. Only patients experiencing a G2‐TLN were included in the voxel‐wise analysis and statistically significant differences between dose‐matched LET_d_ distributions within necrotic‐brain areas (necrosis contoured at onset) and healthy‐brain voxels were explored through association tests and mixed‐effects logistic regression models within dose bins. On a structure‐wise level, G2‐TLN association with clinical and DLVH variables was analyzed for each temporal lobe to implement a pre‐processing pipeline. Hence, bootstrap enhanced Elastic‐Net regularized logistic regression, followed by the area under the receiver operating characteristic curve (AUROC), was used to select the final model. Performance and calibration were evaluated with cross‐validation AUROC and Hosmer‐Lemeshow test, respectively.

**Results:**

With a median follow‐up of 49.7 months, 13 patients experienced G2‐TLN (17 TL) after a median time of 21.5 months. Seven out of 13 G2‐TLN patients reported a statistically significant difference between LET_d_ in necrotic‐ and healthy‐brain voxels after dose‐matching, with 71.4% of them presenting higher LET_d_ values in necrotic‐brain voxels. LET_d_ showed significant associations with the onset of G2‐TLN in dose bins 35–40 Gy(RBE) (Odds Ratio, OR: 7.4, 95%CI: 3.5–26.5, *p* < 0.001) and 50–55 Gy(RBE) (OR: 2.4, 95%CI: 1.5–4.0, *p* < 0.001). Regarding structure‐wise modelling, the final logistic regression model was built using two DLVH variables, V_(dose,LETd)_ (V_(68,0)_ and V_(19,4.6)_), which showed an independent association with G2‐TLN and proved the highest AUROC. Good performance and calibration were reported by the cross‐validation AUROC (0.89, 95%CI: 0.78–0.95) and Hosmer‐Lemeshow test (*p* = 1).

**Conclusions:**

Voxels receiving higher LET_d_ in the low and medium dose ranges (35–40 and 50–55 Gy(RBE)) were associated with high‐grade necrosis on a voxel‐wise level. The volumes of TL receiving high doses (68 Gy(RBE)) or high LET_d_ (4.6 keV/µm) with doses higher than 19 Gy(RBE) were the major predictors of G2‐TLN. Further evaluation with a larger sample size and an external validation cohort is necessary to assess our findings and validate the presented model.

## 1 INTRODUCTION

Temporal lobe necrosis (TLN) is one of the most severe late toxicities for skull‐base and nasopharyngeal treatments after radiotherapy (RT).^1^, ^2^ Clival or petroclival tumors, like chordoma and chondrosarcoma, abutting or compressing one or both temporal lobes, are routinely at risk of receiving doses close to the prescription, and tissue sparing might not always be possible without affecting target coverage and local control.

Due to protons’ physical selectivity with a sharp increase of the dose in a well‐defined depth and a rapid dose fall‐off distal to the tumor, proton therapy (PT) is considered one of the most effective radiation modalities for the management of these tumours.^3^, ^4^, ^5^, ^6^ Moreover, protons are characterized by a favorable radiobiological effectiveness (RBE) compared to photons. In‐vivo and in‐vitro experiments have reported a variability in the RBE mainly due to the variation of Linear Energy Transfer (LET) along the proton beam path, coupled with the peculiarities in tissues’ radiosensitivity.^7^, ^8^, ^9^ However a recent European survey reported that all the surveyed proton therapy centers used a constant RBE of 1.1 in clinical practice, while addressing the variable RBE by including geometrical constraints to avoid beams stopping in the close nearby or within Organs At Risk (OARs).^10^, ^11^ Nonetheless, a recent study on brain tumors highlighted sub‐optimal toxicity outcomes due to variable RBE^12^. Heuchel et al. highlighted the importance of gathering clinical evidence from retrospective and prospective studies investigating the actual role of variable RBE, and the dose‐averaged LET (LET_d_) was reported as the most commonly used variable‐RBE surrogate to overcome the uncertainty of RBE models.^10^, ^13^, ^14^ To date, while the incidence and severity of radionecrosis were established as dose‐volume dependent, with high doses to a small volume being the main causal factor, the detrimental role of LET_d_ in the TLN remains unclear and was not deeply explored.^15^ In brain tumors, on a voxel‐wise level, an overall high heterogeneity across patients was reported, and the conclusions on the marginal role of LET_d_ in determining brain necrosis after proton therapy were controversial.^16^, ^17^, ^18^ On a patient‐wise level, novel dose‐ LET_d_ ‐volume cumulative histogram (DLVH) approaches were evaluated to build normal tissue complication probability (NTCP) models, as reported in a recent head and neck review.^19^ Nonetheless, such promising methods were not explored for brain tumors.

Our study aimed to investigate and model on voxel‐wise and structure‐wise levels the combined effect of dose and LET_d_ in the development of high‐grade temporal lobe radionecrosis, coupled with key clinical variables, in skull‐base PT patients. In this scenario a novel DLVH‐based NTCP model was proposed.

## 2 METHODS

### 2.1 Patient cohort

We included in this retrospective study 79 patients affected by skull‐base chordoma (*N* = 61) and chondrosarcoma (*N* = 18), consecutively treated with PT between 2011 and 2020 at CNAO National Center for Oncological Hadrontherapy (Pavia, Italy) and satisfying the following inclusion criteria: histological diagnosis of skull‐base chordoma or chondrosarcoma, PT as curative intent, at least 9 months of follow‐up.

Skull‐base patients underwent magnetic resonance imaging (MRI) and clinical follow‐up examinations every 3–4, 6, and 12 months in the first 3 years after treatment end, from 3 to 5 years, and afterwards, respectively. The local ethics committee approved this retrospective study (CNAO OSS 38/2021) and all patients accordingly provided written informed consent. This retrospective study was based on clinical data collected in the CNAO Institutional Observational Registry.

### 2.2 PT planning and delivery

Details on target volume delineation, PT planning and delivery procedures have been previously reported.^3^, ^4^ Briefly, all patients were treated in supine position with a customized thermoplastic head‐mask and mouth‐bite. Before treatment, a simulation computed tomography (CT) and MRI scans were acquired in the same setup condition to aid target volumes and OARs segmentation, according to contouring guidelines.^20^

**FIGURE 1:**
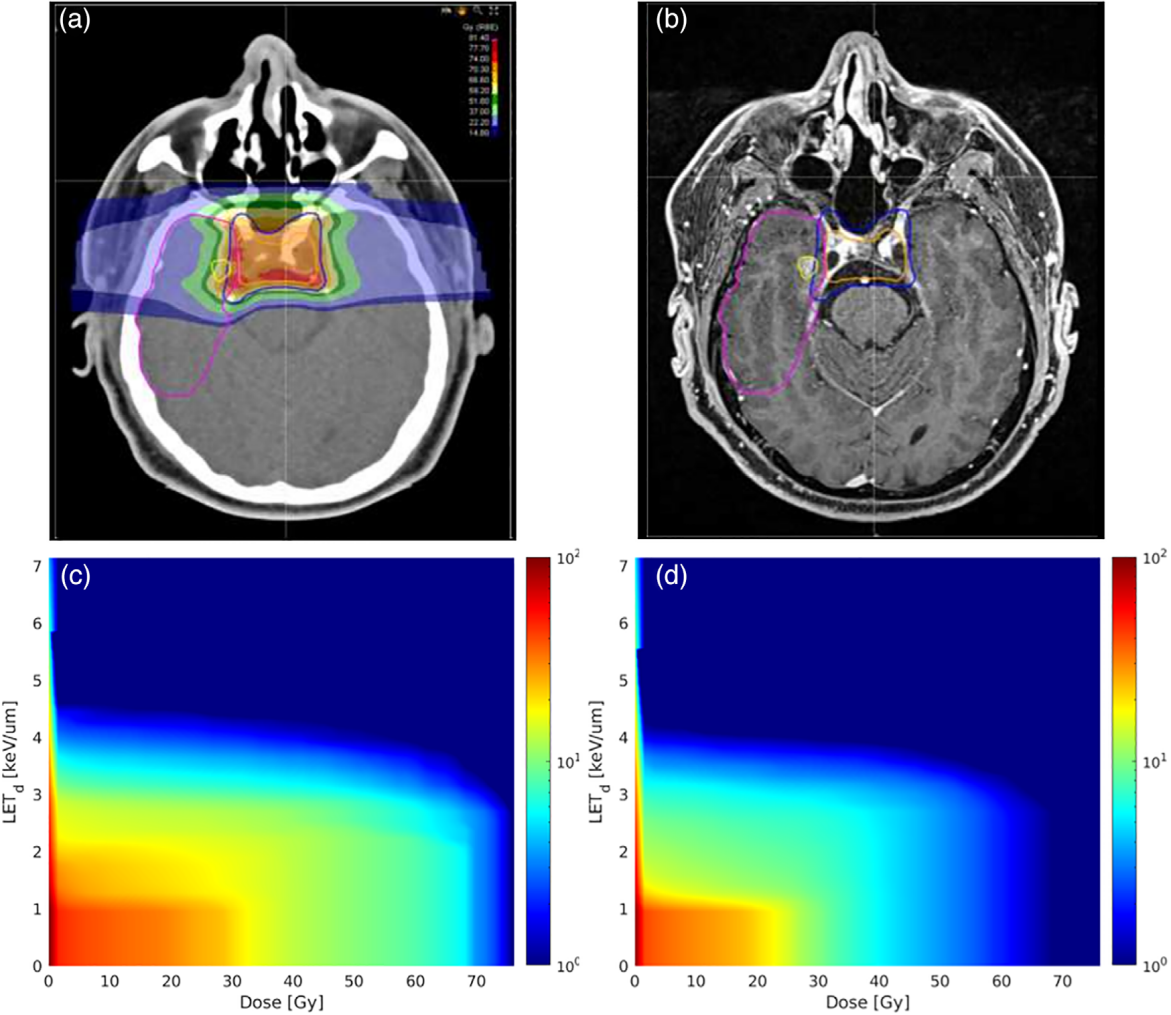
(a)Simulation computed tomography and radiotherapy dose maps, (b) T1‐weighted magnetic resonance imaging sequence with temporal lobe (purple), target (gross tumor volume: orange, clinical target volume: blue) and radionecrosis (yellow) segmentations, (c) median dose‐ LET_d_ cumulative histogram for the temporal lobe in the G2‐TLN patients’ group, (d) median dose‐ LET_d_ cumulative histogram for temporal lobe in the control group.

A total dose of 74 Gy(RBE) for chordomas and 70 Gy(RBE) for chondrosarcomas was delivered (5 daily fractions/week) following a sequential boost scheme with target shrinkage after 27 fractions (54 Gy(RBE)). All patients were treated with pencil beam scanning (PBS), on a fixed horizontal proton beamline using two opposing fields (about 95% of cases) or three fields (5%) (Figure 1a). Two orthogonal X‐rays acquisitions, with automatic rigid registration software and 6‐degree of freedom robotic patient positioning system were used for daily patient setup verification and correction.^21^ RBE‐weighted dose was calculated setting a constant value of 1.1 and TL dose was constrained to D2cc < 71 Gy(RBE) in the optimization process.^22^, ^23^

The patient collection covered almost over 10 years, during which two different treatment planning systems (TPS) were used. Namely, in 2018 CNAO moved from the Syngo v.C13 TPS (Siemens, Erlangen, Germany) to RayStation (RaySearch, Stockholm, Sweden). In the framework of this study, 62 (78.5%) patients were treated with Syngo‐optimized treatment plans and 17 (21.5%) with RayStation‐optimized treatment plans. To guarantee the consistency of the analysis, all plans were recalculated with the Monte Carlo RayStation engine.

### 2.3 Definition of toxicity, clinical and DLVH parameters

TLN was identified on the follow‐up MRI (Figure 1b) and scored using the Common Terminology Criteria for Adverse Events version 5.0 (CTCAE) scale.^24^ High‐grade TLN was defined as CTCAE grade ≥ G2 (G2‐TLN). The following clinical data were collected: age at treatment start, gender, number and surgery technique (endoscopy only or craniotomy), concomitant diseases (diabetes and hypertension).

To evaluate the combined effect of biological dose and LET_d_, the cumulative DLVH was computed as described by Yang et al.^25^ Specifically, 2254 DLVH parameters were considered in our analysis, homogeneously sampled in the [0, 76.19] Gy(RBE) dose and [0, 7.14] keV/um LETd ranges with a 49x46 binning. While being substantially consistent with dose and LET_d_ per bin,^25^ such binning choice represented the optimal trade‐off between DLVH resolution, redundancy and stability.

### 2.4 Statistical analysis

Median and inter‐quartile range (IQR), as well as counts and percentages, were used to describe quantitative and categorical variables. G2‐TLN incidence rate was computed, in addition to G2‐TLN free survival probability, according to the Kaplan‐Meier method. The 95% confidence interval (95%CI) was provided. A significance level equal to 0.05 was considered throughout the study.

### 2.5 Voxel‐wise investigation of dose and LET_d_ relationship

Only skull‐base patients with a reported G2‐TLN after PT were investigated in the voxel‐wise analysis. TLN was segmented on the post‐contrast T1‐weighted MR sequence that proved the toxicity onset. The brain, TLN, and low‐risk clinical target volume (CTV) structures were retrieved, along with dose and LET_d_ maps. Therefore, we investigated the dose‐LET_d_ relationship of healthy‐brain and necrotic‐brain voxels outside the low‐risk CTV. A greedy random matching (GRM) (caliper = 0.1) was implemented to match healthy‐brain with necrotic voxels based on the corresponding doses.^16^ Statistically significant differences in the necrotic and healthy‐brain dose‐matched LET_d_ were explored both within the whole dose distribution and within 5 Gy(RBE) dose bins. Dose binning was implemented to provide insights on the peculiar impact of LET_d_ at different dose levels, with 5 Gy(RBE) identified as a trade‐off between the bin sample size and the need for fine‐grained information. Finally, within each dose bins and with respect to dose matched LET_d_ voxels, logistic mixed‐effects models were fitted to account for individual variability. Random intercept (RI) models were preferred unless the increased complexity of introducing random slopes (RS) was justified by a significantly improved model performance (likelihood ratio test, *p* < 0.05). In instances where the random intercept variance was estimated at zero, the model was treated as a standard logistic regression for the purpose of inference. To evaluate the association between LET_d_ and G2‐TLN, we reported fixed‐effect odds ratios (OR) with 95% confidence intervals (95% CI), associated *p*‐values, and marginal *R* ^2^ values. Additionally, the area under the receiver operating characteristic curve (AUROC) was computed to assess the discriminative performance. To ensure comparability across all models, AUROCs were calculated based on fixed‐effect predictions only. Indeed, our voxel‐wise analysis was limited by a small sample size hence it was designed for inferential purposes instead of predictive modelling. All the voxel‐wise statistical analyses were performed with R studio 4.4.0.^26^

### 2.6 Structure‐wise TLN modelling

Clinical variables preselection was based on the association of all variables (standardized if quantitative) with G2‐TLN as investigated with chi‐squared or Fisher's Exact test, and t‐ or Mann‐Whitney U test, respectively for categorical and quantitative variables (*p* < 0.1). On the other hand, since variables related to dose and LET_d_ were sampled from TL DLVH, a relevant amount of intrinsic information redundancy was expected. Hence, a combined correlation‐ and rank‐based preselection procedure was preferred over plain association tests. Briefly, in the pre‐selection process DLVH variables with negligible informative content were first excluded (variance < 10^−6^), then a Spearman correlation matrix, ordered by Mann‐Whitney U test‐based ranking, was computed to exclude highly correlated variables (rho > 0.7).

Subsequently to select the most robust set of DLVH variables, the pre‐selected variables were fed as input to a logistic regression least squares penalized method, with bootstrap enhanced Elastic‐Net (BE‐E‐Net). This approach was adopted for its reported robustness to redundancy compared to its closest variant, the least absolute shrinkage and selection operator (LASSO).^27^, ^28^ 2000 bootstrap samples were generated to ensure the numerical stability and convergence of variable inclusion probabilities, thereby minimizing the Monte Carlo sampling error.^29^, ^30^ For each bootstrap sample, the predictors selected by the Elastic‐Net penalized logistic regression model were recorded, along with their coefficients and the model Bayesian and Akaike Information Criteria (BIC and AIC). These metrics were chosen to represent the balance between the goodness‐of‐fit and structural complexity, where relatively lower values are associated with the optimal model. The number of predictors (*N*) mostly selected across the bootstrap samples while minimizing both the associated AIC and BIC values was identified. Finally, the optimal set of *N* independent predictors was selected in order to maximize the model accuracy, as measured by the AUROC, while ensuring no variable multicollinearity. The classification performance of the final non‐penalized logistic regression model was assessed via 10‐fold cross‐validation (cv). The AUROC was evaluated along with its 95% percentile bootstrap CI (*n* = 1000). Statistical significance was determined via permutation testing (*n* = 1000 permutation samples), to compare the observed performance against a null distribution. Finally, the model calibration—the agreement between the observed and the predicted probabilities—was assessed using the calibration plot and the Hosmer‐Lemeshow test (HL).

All the methodological details were provided as the Supplementary Materials (NTCP modeling pipeline S‐1). NTCP modelling analyses were performed with Matlab R2022b.^31^

## 3 RESULTS

The main patient, disease and treatment characteristics are reported in Table 1. With a median follow‐up of 49.7 months, 30 (38%) patients, out of 79, developed TLN of any grade. No case of toxicity ≥G3 was registered, while G2‐TLN was reported in 13 (16.5%) patients, corresponding to a total of 17 structure‐wise G2‐TLN events (i.e., incidence rate of 10.8%; 95% CI: 5.9%–15.6%). In 9 (69%) cases TLN affected only one temporal lobe, whereas in 4 (31%) cases TLN was bilateral. The median time to G2‐TLN was 21.5 months, with a 2‐year G2‐TLN free survival probability of 89.2% (Figure S1). The median G2‐TLN volume was 0.14 cc [0.08 cc; 0.40 cc], with a median [IQR] dose and LET_d_ of 66.04 [64.24; 73.57] Gy(RBE) and 3.53 [3.20; 3.68] keV/µm, respectively.

**TABLE 1:** Patient, disease, and treatment characteristics according to grade ≥2 temporal lobe necrosis status.

		Temporal lobe necrosis ≥ G2		
	Overall, *N* = 79^1^	No, *N* = 66^1^	Yes, *N* = 13^1^	*p*‐value^2^
**Age [years]**	57.5 (48.2, 67.4)	57.5 (48.6, 67.4)	59.8 (43.1, 66.0)	0.890
**Gender**				0.390
Female	40 (51%)	32 (48%)	8 (62%)	
Male	39 (49%)	34 (52%)	5 (38%)	
**Histology**				0.159
Chondrosarcoma	18 (23%)	13 (20%)	5 (38%)	
Cordoma	61 (77%)	53 (80%)	8 (62%)	
**Surgery**	77 (97%)	65 (98%)	12 (92%)	0.304
**Total number of surgeries**	1.0 (1.0, 2.0)	1.0 (1.0, 2.0)	1.0 (1.0, 1.0)	0.543
**Surgical technique**				>0.999
Craniotomy	16 (20%)	13 (20%)	3 (23%)	
Endoscopic endonasal approach	61 (77%)	51 (77%)	10 (77%)	
Endoscopic endonasal approach + craniotomy	2 (2.5%)	2 (3.0%)	0 (0%)	
**Hypertension**	25 (32%)	21 (32%)	4 (31%)	>0.999
**Diabetes**	4 (5.1%)	4 (6.1%)	0 (0%)	>0.999
**Baseline_macroscopic_disease**	63 (80%)	52 (79%)	11 (85%)	>0.999
**GTV [cc]**	6.5 (1.1, 15.9)	7.0 (1.5, 15.0)	4.9 (0.9, 16.2)	0.773
**PT Intent**				0.188
Adjuvant PT	74 (94%)	63 (95%)	11 (85%)	
Exclusive PT	5 (6.3%)	3 (4.5%)	2 (15%)	

*Note*: Clinical and treatment characteristics for the overall cohort and stratified by grade ≥2 temporal lobe necrosis (TLN) status. Data are presented as median [IQR] or *n* (%). *P*‐values refer to comparisons between patients with and without grade ≥2 TLN.

^1^ Median (IQR); *n* (%).

^2^ Wilcoxon rank sum test; Pearson's Chi‐squared test; Fisher's exact test.

### 3.1 Voxel‐wise investigation of dose and LET_d_ relationship

**FIGURE 2:**
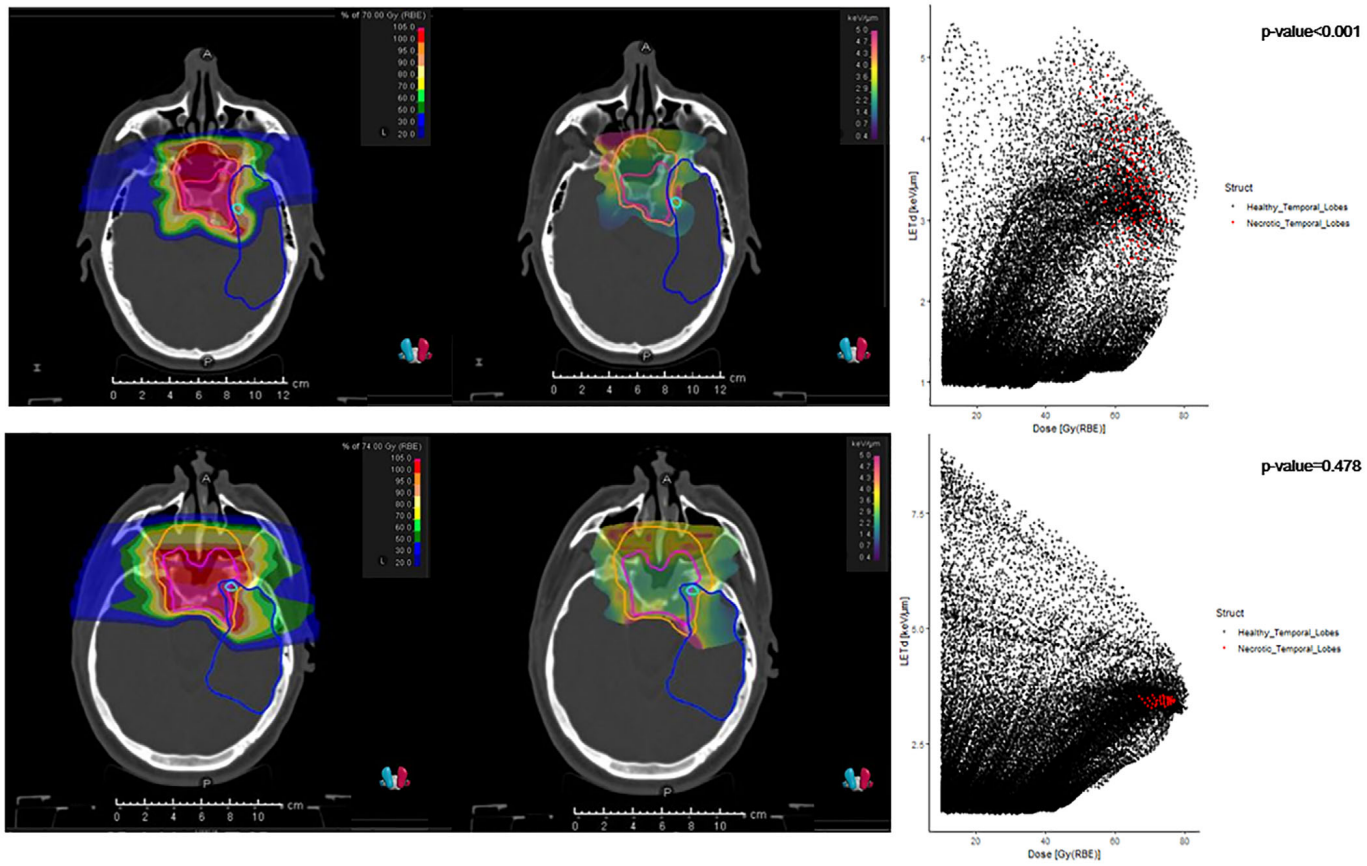
Dose maps, LET_d_ maps and voxel‐wise distributions for necrotic and healthy temporal lobe tissue. Left and central panels show dose and LET_d_ maps overlaid on the simulation CT, respectively. Right panels show voxel‐wise dose‐LET_d_ distributions, with necrotic voxels displayed in red and healthy‐brain voxels in black. *P*‐values were derived from the Mann‐Whitney *U* test. Two representative patient cases are shown.

Seven out of 13 G2‐TLN patients reported a statistically significant difference between LET_d_ in necrotic‐brain and healthy‐brain voxels after dose‐based GRM, with 71.4% of them presenting higher LET_d_ distribution in necrotic‐brain voxels (Figure 2).

Full patient‐based results for the whole dose distribution were provided in Table S1. With respect to all G2‐TLN patients’ data, significant differences were measured for all evaluated dose bins except for 45–50 and 65–75 Gy(RBE). For each dose bins, delta LET_d_ values were reported in Figure S2 describing a decreasing trend in delta LET_d_ with increasing doses. Nonetheless, it is worth noticing that only few voxels belonging to one and two patients reported a necrosis in the dose bins 25–35 and 35–40 Gy(RBE), respectively.

Dose‐matched LET_d_ also showed significant associations in dose bins 35–40 Gy(RBE) (Odds Ratio, OR: 7.4, 95%CI: 3.5–26.5, *p* < 0.001) and 50–55 Gy(RBE) (OR: 2.4, 95%CI: 1.5–4.0, *p* < 0.001) through RI‐only model (with RI standard deviation ∼ 0). In both cases, the baseline risk of developing a G2‐TLN within the specific dose bin was significantly low as measured by the respective intercept estimates (OR = 0.003, 95%CI: 0–0.3, *p* < 0.001 and OR = 0.05, 95%CI: 0.01–0.24, *p* < 0.001). The computed AUROC was good for 35–40 Gy(RBE) dose bin (0.85, 95%CI: 0.77–0.94), while fair for the 50–55 Gy(RBE) dose bin (0.67, 95%CI: 0.59–0.76). However, caution should be kept given the small sample size characterizing especially the 35–40 Gy(RBE) dose bin. Of note, poor AUROC values were observed for dose bins above 55 Gy(RBE). RS models were selected in the following dose intervals: 40–45 Gy(RBE), and 55–70 Gy(RBE), however the former model did not converge hence results were not presented. In these dose intervals, no significant associations were observed between LET_d_ and G2‐TLN; furthermore, these models were characterized by negligible marginal R^2^ values, indicating that LET_d_ explained very little of the outcome variance. Table 2 summarizes the computed model metrics.

**TABLE 2:** Dose‐binned LET_d_ distributions and logistic mixed‐effects model's parameters and evaluation metrics.

				Logistic mixed‐effects models			
Dose bin [Gy(RBE)]	Necrotic‐brain LET_d_ [kev/um]^1^	Healthy‐brain LET_d_ [kev/um]^1^	*p*‐value ^2^	LET_d_ OR (95% CI)^3^	*p*‐value ^3^	Marginal R^2^	AUROC^4^ (95% CI)
25–30	4.36 [1.15]	1.56 [0.99]	<0.001	–	–	–	–
30–35	3.31 [1.64]	1.77 [0.46]	<0.001	–	–	–	–
35–40	3.24 [1.17]	2.12 [0.73]	<0.001^*^	7.37 (3.47; 26.52)	<0.001	0.52	0.86 (0.77; 0.94)
40–45	3.62 [1.14]	2.71 [0.91]	<0.001	^‐§^	^‐§^	^‐§^	^‐§^
45–50	3.38 [0.78]	3.17 [0.73]	0.145	1.35 (0.81; 2.25)	0.230	0.02	0.58 (0.47; 0.70)
50–55	3.89 [0.80]	3.34 [0.94]	<0.001	2.39 (1.51; 3.98)	<0.001	0.11	0.67 (0.59; 0.76)
55–60	3.80 [1.35]	3.38 [0.99]	0.001	2.09 (0.18; 21.20)^§^	0.528^§^	0.03^§^	0.59 (0.54; 0.65)^§^
60–65	3.04 [1.27]	3.04 [1.45]	0.021	1.10 (0.10; 10.43)^§^	0.925^§^	<0.01^§^	0.45 (0.40; 0.49)^§^
65–70	3.19 [1.21]	3.07 [1.41]	0.174	1.41 (0.36; 5.50)^§^	0.606^§^	0.01^§^	0.53 (0.49; 0.56)^§^
70–75	3.53 [0.58]	3.55 [0.65]	0.841	1.09 (0.86; 1.44)	0.527	<0.01	0.50 (0.46; 0.55)

*Note*: Voxel‐wise dose‐binned LET_d_ distributions after dose‐based greedy random matching. Data are presented as median [IQR] for necrotic and healthy‐brain voxels. Odds ratios (ORs), *p*‐values, marginal *R* ^2^, and AUROC values from logistic mixed‐effects models are reported for each dose bin.

^1^ Median [inter‐quartile range].

^2^ p‐values computed with Mann‐Whitney *U* test.

* p‐value computed with *t*‐test.

^3^ Fixed effect Odds‐Ratio (OR) with 95% bootstrap confidence interval (CI) and *p*‐value.

^§^ models fitted with both random intercept and slope.

^‐§^ model metrics not reported due to convergence issues.

^4^ Area under the receiver operating characteristic curves were computed based only on fixed‐effects.

### 3.2 Structure‐wise TLN modelling

Based on the reported association between the evaluated variables and G2‐TLN (Table 1), all the clinical variables were excluded from the following BE‐E‐Net procedure. Figure 1c,d showed the median DLVH for the two patient subsets (G2‐TLN vs. controls).

After the DLVH pre‐selection filtering based on correlation analysis (more details in NTCP modeling pipeline S‐1), 13 DLVH variables were considered. The full list of the DLVH variables selected used as input of the BE‐E‐Net selection approach is reported in Figure S3a. As a result, the BE‐E‐Net procedure selected the models based on two variables, as locally minimizing both AIC and BIC (Figure S3b), hence they were evaluated further to implement the final model. To build the final model, the V_(dose, LETd)_ variables selected more than 50% times in the BE‐E‐Net analysis were: (V_(68,0)_, V_(74.6,0)_ and V_(19,4.6)_). Figure S3b reported the selection frequency of all the variables evaluated in the BE‐E‐Net procedure. Given the non‐multicollinearity of the selected variables, the three two‐variable models were fitted and evaluated based on the AUROC.

Table 3 described the non‐penalized final model, with the two selected variables (V_(68,0)_ and V_(19,4.6)_), while the other fitted models were reported in Table S2.

**TABLE 3:** Final multivariable logistic regression model for prediction of grade ≥2 temporal lobe necrosis.

	Estimate	95%CI	*p*‐value	AIC	BIC	AUROC	95%CI	Calibration slope	HL (*p*‐value)
**Intercept**	−4.53	−5.98; −3.08	<0.001	74	83	0.89	0.78; 0.95	1.06 (< 0.001)	0.071 (1)
**V_(68,0.0)_**	0.60	0.33; 0.87	<0.001						
**V_(19,4.6)_**	1.33	0.16; 2.50	0.02						

*Note*: Coefficient estimates with corresponding 95% confidence intervals (CIs) and *p*‐values for the final multivariable logistic regression model predicting grade ≥2 temporal lobe necrosis (TLN). Model performance and calibration is described by the Akaike information criterion (AIC), Bayesian information criterion (BIC), area under the receiver operating characteristic curve (AUROC) with 95% CI, calibration slope, and Hosmer–Lemeshow goodness‐of‐fit test.

**FIGURE 3:**
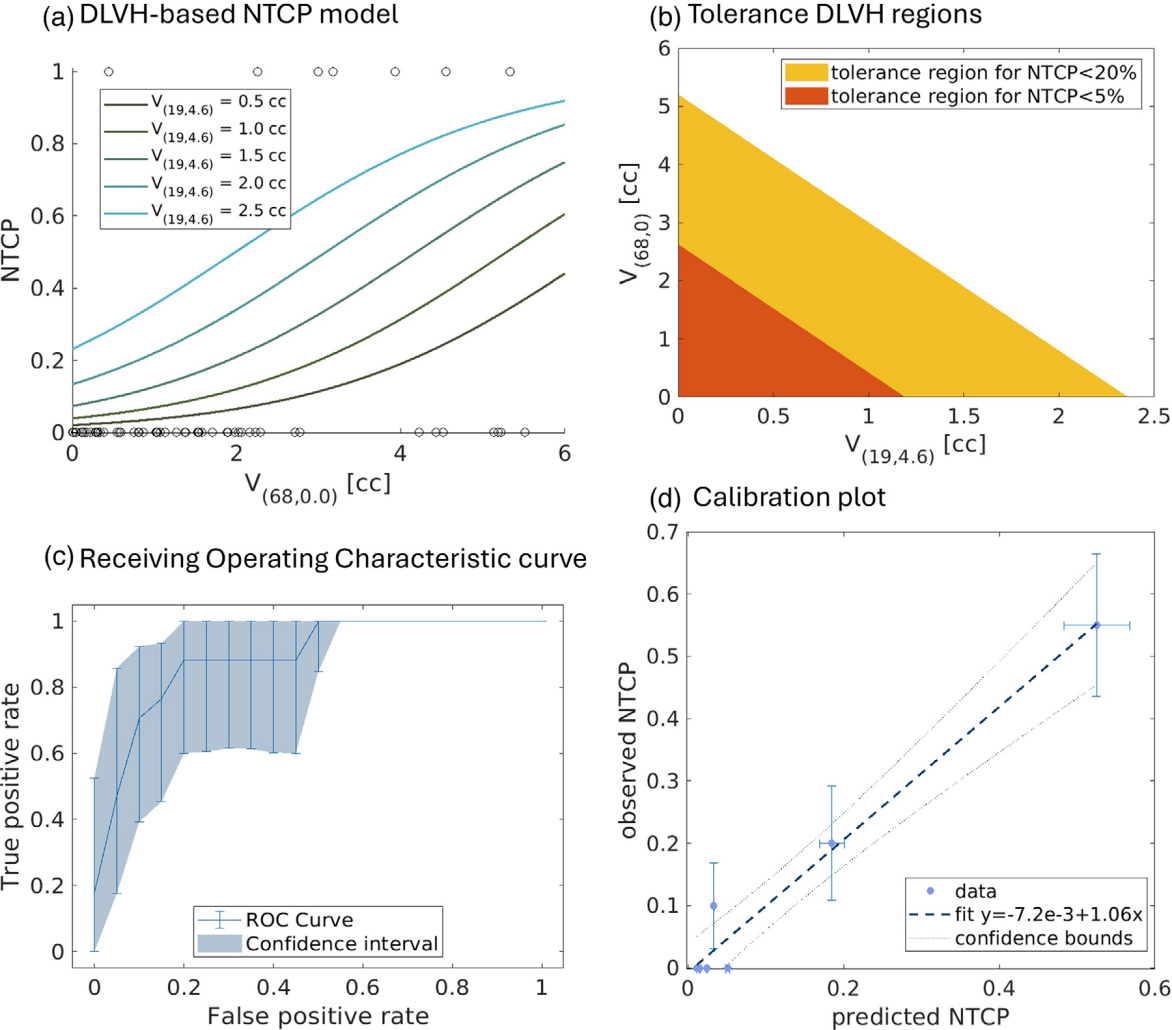
(a)Final DLVH‐based NTCP model. DLVH‐based NTCP model presented with respect to both the selected DLVH variables (b) DLVH tolerance regions corresponding to 5% and 20% G2‐TLN probability (c) receiver operating characteristic curve with point‐wise 95% confidence intervals (d) and model calibration plot with linear regression and confidence bounds.

Figure 3 reported the final DLVH‐based NTCP model and the relation between the two dosimetric variables (Figure 3a), highlighting the combined values associated with a G2‐TLN probability lower than 5% and 20% (Figure 3b).

The cv‐AUROC for the non‐penalized NTCP final model was 0.89 (95% bootstrap CI: 0.78–0.95), and was proved to be statistically significant at the permutation test (*p* < 0.001). The ROC curve, with point‐wise confidence intervals, was computed and shown in Figure 3c. Moreover, the calibration plot with the estimated linear regression coefficients highlighted a good agreement between observed and predicted probabilities (Figure 3d, *p* = 1).

## 4 DISCUSSION

Our study explored the combined effect of LET_d_ and absorbed dose on the likelihood of developing a high‐grade TLN in a homogeneous cohort of chordoma and chondrosarcoma patients treated with proton therapy. Both voxel‐wise and structure‐wise analyses were performed for a comprehensive view of the paired absolute and cumulative impact of such relevant metrics, in the attempt to address the increasing need for retrospective studies on the matter.^10^

The current series reports an incidence of grade 2 TLN of 16.5% of the patients, higher than the rates described in other proton series.^22^, ^32^, ^33^, ^34^ A large cohort study by Schroder et al^32^ reported a percentage of G2‐TLN of 9%, with a median follow‐up of 51 months. A possible explanation for the lower rate, compared to our series, may be due to the inclusion of heterogeneous histologies (61.5% skull‐base chordomas) and both adult and pediatric patients, resulting in a lower mean age (45 years old). Since that study found a stronger correlation between the incidence of G2‐TLN and age, it supports the hypothesis that more unfavorable clinical parameters in our series, such as a higher median age (57 years) and the inclusion of chordoma and chondrosarcoma histologies only, could contribute to the higher observed rate. Furthermore, in our series 78.5% of patients were treated with Syngo‐optimized plans that underestimated the actual delivered doses. A detailed analysis by Molinelli et al. pointed out that, for head‐and‐neck treatments, the Syngo pencil beam algorithm underestimated near‐to‐maximum doses to the target by 7% on average with respect to the Monte Carlo (MC) dose engine used in RayStation.^35^ Syngo‐based planning coupled with sub‐optimal fixed‐beam delivery geometry has likely contributed to unfavorable D_RBE_ distribution. Previous series on central nervous system tumors reported that a higher number of treatment fields allows not only for a more conformed dose deposition, but also for a better distribution of high LET_d_ regions.^17^, ^36^ At our institution, MC calculation engines are now routinely adopted for proton planning and skull‐base chordoma and chondrosarcoma are preferably planned with a three to four‐field configuration.^37^

In our series at the voxel‐wise level, 54% of the patients affected by G2‐TLN showed significantly different LET_d_ distributions between necrotic‐ and healthy‐brain voxels, with higher LET_d_ values within the necrosis in 5 cases (38.5% of all G2‐TLN patients). Even though the LET_d_ values were evaluated within a uniform dose distribution (GRM), these results suggested that no strong absolute relationship between LET_d_ and TLN exists. However, we recorded peculiar LET_d_ behaviors across the explored 5 Gy(RBE)‐dose bins. In particular, a significant trend of decreasing differences between median LET_d_ in necrotic and healthy brain voxels with increasing matched doses was observed (Figure S2). This pattern supports the hypothesis of a more relevant LET_d_ contribution at lower doses, while a negligible LET_d_ effect at higher doses. From a radiobiological perspective, this behavior is consistent with the expectation that LET‐driven RBE variations become more relevant in the low‐to‐intermediate dose range, whereas at high doses the dominant effect of physical dose may mask LET‐dependent variations. Similarly, Bertolet et al. and Vestergaard et al. reported LET_d_ as a robust predictor for brain radionecrosis after proton therapy while acknowledging a masking effect at high doses.^17^, ^36^ The latter study also confirmed previous investigations by Bahn et al. and Eulitz et al. in glioma cohorts, identifying the interaction between dose and LET_d_, together with periventricular distance, as robust predictors for brain image changes after proton therapy.^38^, ^39^ Specifically, Bahn et al. concluded that RBE increases with radiation quality, as reflected by LET_d_, and developed a predictive logistic regression model with high discriminative performance (AUROC = 0.94) for localizing brain contrast‐enhancing lesions, although without accounting for inter‐patient variability.^38^ In our study, an explicit interaction term between dose and LET_d_ was not included in the regression models, but their interaction was implicitly explored through the dose‐binning and dose‐matching strategy. In this context, the different impact of LET_d_ across dose bins can be interpreted as an interaction between the two dosimetric parameters.

In our voxel‐level analysis, a potential role of LET_d_ emerged particularly at intermediate dose levels (dose bins 35–40 and 50–55 Gy(RBE)). However, only the model fitted within the 35–40 Gy(RBE) bin achieved good discriminative performance (AUROC = 0.85). Yang et al. highlighted the importance of focusing on specific voxel subsets to better model the biological processes underlying radiation‐induced image changes after proton therapy.^25^ In our series, to partially address this limitation, radionecrosis regions were contoured at their first appearance on follow‐up images. Nevertheless, the median lesion size (0.140 cc) was similar to the threshold reported by Bahn et al. (0.135 cc),^38^ and we acknowledge that, beyond the limited statistics, such small volumes may introduce statistical noise at the voxel level, potentially masking the association between LET_d_ and G2‐TLN across dose bins. In line with these findings, Garbacz et al., despite observing significant differences between constant RBE (1.1) and variable RBE dose distributions, reported no clear voxel‐wise association between high LET_d_ and brain radionecrosis in a skull‐base tumor cohort.^40^ Similarly, Niemierko et al. evaluated LET_d_ effects in a large cohort of head and neck and central nervous system patients and found no consistent evidence of a direct role of LET_d_ in brain radionecrosis, possibly reflecting substantial inter‐patient heterogeneity and variability in radiosensitivity.^16^ Compared with whole‐population voxel studies, our dose‐binning approach resulted in lower statistics but was less affected by the large inter‐patient heterogeneity reported in previous studies.^16^, ^17^, ^36^, ^40^ At the same time, the dose‐binning strategy required substantially lower computational effort while allowing a simplified interpretation of model outcomes and facilitating the identification of dose‐specific LET_d_ behaviors that might remain hidden in analyses performed across the full dose range. Voxel‐wise modelling nevertheless presents several methodological limitations, including the lack of volume‐effect, statistical noise due to the inclusion of a large number of non‐specific voxels, the unrealistic assumption of voxel independence (particularly within the same patient or necrotic region), and the difficulty of retrospectively identifying the biological seed regions of necrosis. Despite these limitations, voxel‐based modelling remains a valuable tool for identifying spatial risk patterns within treatment plans and may support the identification of high‐risk regions potentially exploitable for adaptive treatment optimization. Moreover, voxel‐based models may also be used to derive global NTCP estimates, as suggested by Bahn et al. in glioma patients.^38^ However, to date, no validated voxel‐wise models are available to identify temporal lobe high‐risk regions for G2‐TLN after proton therapy, nor is a globally accepted structure‐wise NTCP model available.

While voxel‐level analyses provide valuable mechanistic insights into the spatial interplay between dose and LET_d_, their translation into routine clinical decision‐making remains challenging due to the lack of validated models and standardized implementation strategies. Voxel‐wise and structure‐wise modelling should be considered complementary rather than competing approaches. Voxel‐based analyses are particularly suited to identifying spatial patterns of complication risk within treatment plans, whereas DLVH‐based models may provide insights into the cumulative joint effect of dose and LET_d_. Both approaches therefore offer complementary tools for treatment plan optimization, either through the identification of high‐risk regions or through the derivation of clinically applicable DLVH‐based constraints.

Heuchel et al. reported that LET_d_ was the most commonly measured metrics over the 25 surveyed European proton centers, while RBE distribution calculation is not yet standardized due to the variety of available RBE models and the inherent lack of consensus.^10^, ^13^, ^14^ In this scenario, a recent review on head and neck proton therapy by Chen et al. endorsed the implementation of DLVH‐based NTCP models as a future strategy to incorporate RBE‐optimized treatment planning into clinical practice. The authors claimed for prospective clinical trials and retrospective analyses aimed at deriving dose‐LET_d_‐volume constraints (DLVC),^19^ particularly now that joint dose‐LET_d_ evaluation has become available in commercial treatment planning systems.

In the present study, we therefore systematically developed a DLVH‐based NTCP model on a structure‐wise basis to predict the likelihood of developing a G2‐TLN after skull‐base proton therapy. V_(68,0)_ and V_(19,4.6)_ emerged as independent predictors of G2‐TLN, while clinical variables were excluded during the pre‐selection process, in contrast to previous literature findings.^6^, ^41^, ^42^ The substantially higher selection frequency of the dose‐volume parameter V_(68,0)_ suggests a predominant role of high doses in inducing G2‐TLN, which is consistent with previously published dose‐volume NTCP models.^32^, ^43^ Indeed, although different variables have been proposed in literature (e.g., D1cc, D2cc^32^, ^33^), our results further emphasize that the delivery of high radiation doses to small volumes plays a key role in determining severe TLN.

At the same time, our model highlighted a potential role of LET_d_ through the parameter V_(19,4.6)_, with a TL volume smaller than 1.19 cc and 2.36 cc receiving at least 19.4 Gy(RBE) and 4.6 keV/um predicting 5% and 20% probabilities of G2‐TLN, respectively. Considering the relatively low dose associated with this DLVH parameter and the large portion of temporal lobe volume it encompasses, the presence of high LET_d_ values may become particularly relevant in determining tissue response. The developed model demonstrated a good performance, calibration, and goodness of fit. However, despite its promising performance, DLVH‐based approaches also present limitations, including the lack of spatial resolution and potential statistical uncertainties at the boundaries of the dosimetric distribution.

To the best of our knowledge, this is the first study evaluating the combined effect of LET_d_ and dose both at voxel‐ and structure‐wise levels in a highly homogeneous cohort of skull‐base patients in terms of prescription dose, treated volume and histology. However, beyond the methodological limitations discussed above, this study is constrained by its retrospective design, potential treatment planning bias, and a relatively limited sample size. Furthermore, external validation is currently lacking and future studies should incorporate additional patient‐specific information (e.g., quality‐of‐life questionnaire, biological markers) and imaging‐based features, which may contribute to modelling inter‐individual radiosensitivity. Finally, the use of LET_d_ as a surrogate for radiation quality has known limitations and represents only an approximation of RBE without fully capturing the complex radiobiological mechanisms described by model‐based RBE calculations. In particular, in treatment scenarios involving opposing beams, LET_d_ may underestimate high‐LET contributions in voxels where the distal edge of one beam overlaps with the entrance region of another beam due to averaging effects. Nevertheless, despite these limitations, LET_d_ is currently the only commercially available parameter that can be incorporated into treatment plan evaluation and optimization to account for radiation quality beyond physical dose.

## 5 CONCLUSIONS

In a homogeneous cohort of chordoma and chondrosarcoma treated with PT, voxels receiving a higher LET_d_ in the low and medium dose ranges (35–40 and 50–55 Gy(RBE)) were associated to high‐grade TLN on a voxel‐wise level. On a structure‐wise level, V_(68,0)_ and V_(19,4.6)_ proved to be independent predictors of G2‐TLN, implementing a well‐performing and calibrated NTCP model. Larger prospective studies and external validation are warranted to confirm and validate our findings and model.

## CONFLICT OF INTEREST STATEMENT

The authors declare no conflicts of interest.

## Supporting information



Supporting Information

Supporting Information

Supporting Information

Supporting Information

Supporting Information
