# Supplementary figures and images for: Modelling dose and dose‐averaged linear energy transfer to predict high‐grade temporal lobe necrosis following skull‐base proton therapy
Source: Med Phys. 2026 Jul 25;53(8):e70562. doi: 10.1002/mp.70562 (PMC13401089; doi:10.1002/mp.70562)

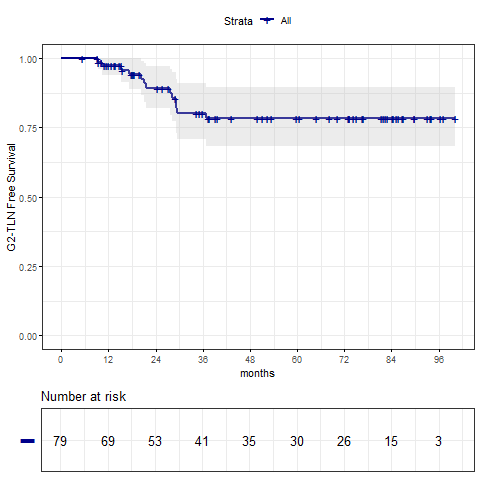

Supplement: Supplementary file 1 — Supporting Information [file MP-53-0-s002.tiff]

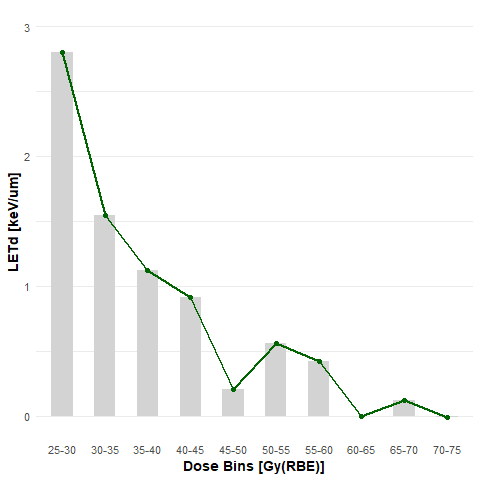

Supplement: Supplementary file 2 — Supporting Information [file MP-53-0-s001.tiff]

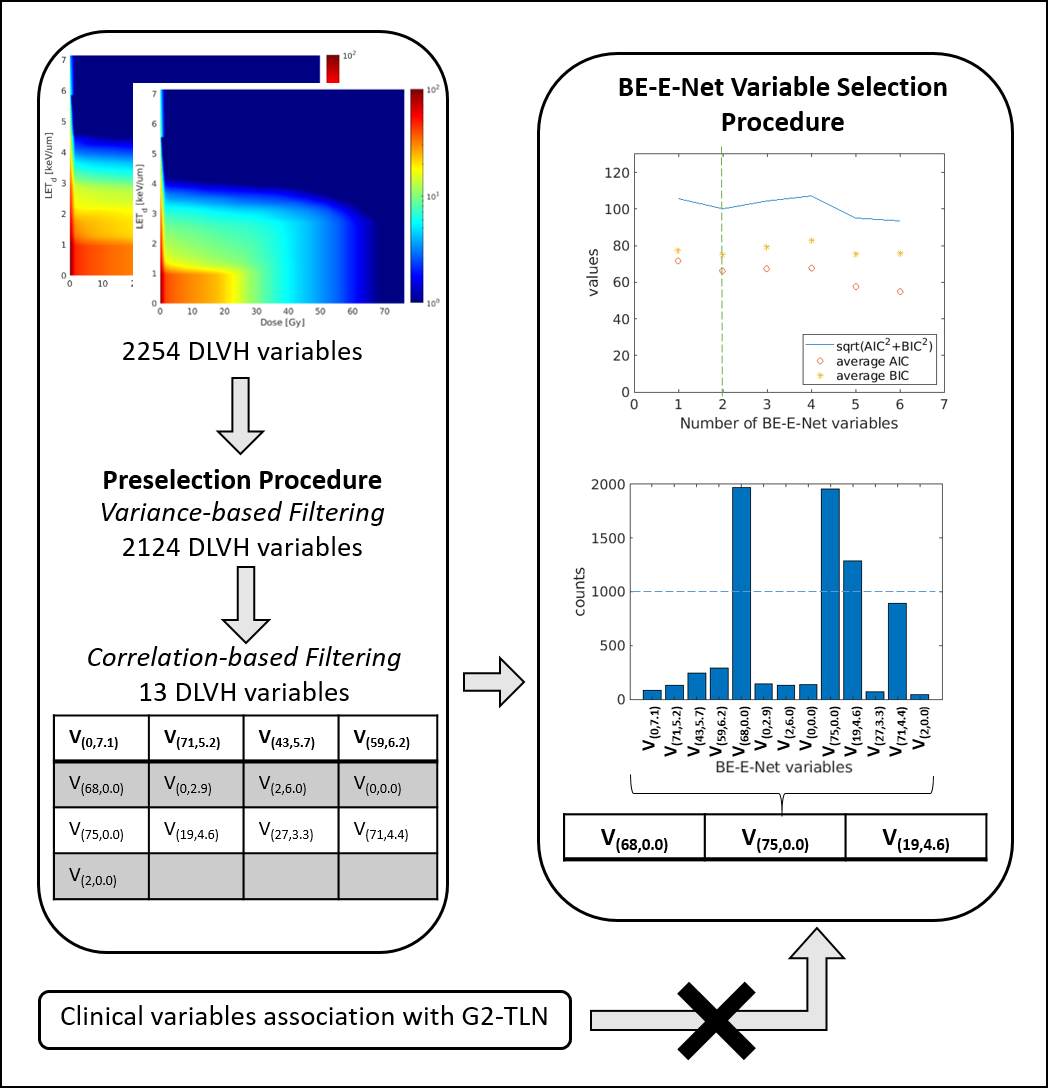

Supplement: Supplementary file 3 — Supporting Information [file MP-53-0-s003.tiff]
