# Supplementary material for: Modelling dose and dose‐averaged linear energy transfer to predict high‐grade temporal lobe necrosis following skull‐base proton therapy
Source: Med Phys. 2026 Jul 25;53(8):e70562. doi: 10.1002/mp.70562 (PMC13401089; doi:10.1002/mp.70562)
Supplement: Supplementary file 4 — Supporting Information [file MP-53-0-s005.docx]

**TABLE S-1** Voxel-wise LET_d_ distributions in necrotic and dose-matched healthy-brain voxels

|  | **Necrotic Brain LETd [keV/um]** | | **Healthy Brain LETd [keV/um]** | |  |
| --- | --- | --- | --- | --- | --- |
|  | **Median** | **IQR** | **Median** | **IQR** | **p-value** |
| *P1* | 4,24 | 0,62 | 3,88 | 0,63 | <0,001 |
| *P2* | 4,10 | 0,23 | 4,02 | 0,44 | 0,06 |
| *P3* | 3,51 | 0,50 | 3,53 | 0,50 | 0,01 |
| *P4* | 1,81 | 0,14 | 1,99 | 0,59 | <0,001 |
| *P5* | 3,80 | 0,67 | 3,33 | 0,78 | 0,09 |
| *P6* | 3,46 | 0,53 | 3,46 | 0,46 | 0,53 |
| *P7* | 4,50 | 0,46 | 4,32 | 0,50 | <0,001 |
| *P8* | 3,41 | 0,30 | 3,41 | 0,56 | 0,41* |
| *P9* | 2,96 | 1,35 | 2,57 | 1,24 | <0,001 |
| *P10* | 3,52 | 0,81 | 2,96 | 1,73 | <0,001 |
| *P11* | 3,43 | 0,09 | 3,44 | 0,38 | 0,48 |
| *P12* | 2,45 | 0,12 | 2,30 | 0,30 | <0,001 |
| *P13* | 2,91 | 0,06 | 2,91 | 0,25 | 0,89 |
| *ALL* | *3,45* | *0,81* | *3,41* | *0,90* | <0,001 |

Median LETd values and interquartile ranges (IQRs) for necrotic and healthy-brain voxels after dose-based greedy random matching. Corresponding p-values are reported for each patient and for the overall cohort. * p-value computed with t-test instead of Mann–Whitney U test.

**TABLE S-2** Alternative multivariable logistic regression models for prediction of grade ≥2 temporal lobe necrosis

|  | **Estimate** | **95%CI** | **p-value** | **AIC** | **BIC** | **AUROC** | **95%CI** |
| --- | --- | --- | --- | --- | --- | --- | --- |
| **intercept** | -4.16 | -5.42; -2.91 | <0.001 | 73 | 82 | 0.888 | 0.75 ; 0.96 |
| **V_(68,0.0)_** | 0.48 | 0.19; 0.78 | 0.001 |  |  |  |  |
| **V_(75,0.0)_** | 0.70 | 0.10; 1.31 | 0.02 |  |  |  |  |

|  | **Estimate** | **95%CI** | **p-value** | **AIC** | **BIC** | **AUROC** | **95%CI** |
| --- | --- | --- | --- | --- | --- | --- | --- |
| **intercept** | -3.54 | -4.57; -2.52 | <0.001 | 81 | 90 | 0.821 | 0.62 ; 0.92 |
| **V_(75,0.0)_** | 1.06 | 0.46; 1.66 | <0.001 |  |  |  |  |
| **V_(19,4.6)_** | 0.96 | -0.25; 2.18 | 0.12 |  |  |  |  |

Resume of the main characteristics of the non-selected evaluated logistic regression models predicting G2-TLN: coefficient estimates, associated 95% confidence intervals and p-value; Akaike and Bayesian Information Criteria; Area Under the Receiver Operating Characteristics curve with the 95% confidence interval.
