# Supplementary material for: Modelling dose and dose‐averaged linear energy transfer to predict high‐grade temporal lobe necrosis following skull‐base proton therapy
Source: Med Phys. 2026 Jul 25;53(8):e70562. doi: 10.1002/mp.70562 (PMC13401089; doi:10.1002/mp.70562)
Supplement: Supplementary file 4 [file MP-53-0-s002.docx]

**S1: Comprehensive NTCP modelling methodological pipeline**

- **Clinical Variable pre-selection**: The association between all standardized clinical variables with G2-TLN was investigated with chi-squared or Fisher Exact test, and t- or Mann-Whitney U test, respectively for categorical and quantitative variables, hence serving as a pre-selection procedure to define potential predictors (p<0.1).

- **Correlation-based pre-selection for the structure-wise analysis**: the DLVH variables with a variance less than 10^-6^ were removed from the analysis due to their reduced informative content in the context of analysis. Then, variable clustering was implemented in order to reduce the number of DLVH variables by considering the most significant and less cross-correlated variables. First, the remaining variables were ranked using Mann-Whitney U test (MWU), hence a MWU rank-ordered cross-correlation matrix was calculated based on Spearman correlation index. Starting from the variable with the highest MWU rank (first row of the correlation matrix), the variables (correlation matrix columns) with a spearman correlation > 0.70 were excluded.

- **Structure-wise NTCP model development:** To obtain the NTCP model from the obtained DLVH variables, the implementation of a logistic regression least squares penalized method with bootstrap enhanced Elastic-Net (BE-E-Net) was preferred over its closest variant, the least absolute shrinkage and selection operator (LASSO) ^1,2^ because of potential redundancy issues. Specifically, -2000 bootstrap samples were derived from our data, classifying patients with healthy TL or TLN<G2 and G2-TLN. For each bootstrap sample, BE-E-Net hyper-parameters (λ and μ) were optimized via 5-fold cross-validation, minimizing the model deviance with a mean squared error metric. Hence, the predictors selected from Elastic-Net penalized logistic regression model were recorded, along with their coefficients and the model Bayesian and Akaike Information Criteria (BIC and AIC). The number of predictors (N) to include in the final model was defined as the mostly selected across the bootstrap samples while minimizing both the associated AIC and BIC values. Each combination of N variables selected more than 50% times were tested for multicollinearity by computing the variance inflation factor (VIF). Therefore, multivariable logistic regression NTCP models were fitted with all the combinations of the N non-collinear (VIF<5) covariates ^3^. The optimal set of N independent predictors was defined based on the AIC criteria, hence the final non-penalized logistic regression model was accordingly implemented.

- **Model classification performance**: 10-fold cross-validation, evaluating the Area Under the Receiver Operating Characteristics Curve (AUROC) along with its 95% bootstrap confidence intervals (1000 bootstrap samples) was used for performance. Furthermore, the cross-validation AUROC statistical significance was assessed with the permutation test, accounting on 1000 permutation samples.

- **Agreement and model calibration:** between the observed and the predicted probabilities, and the goodness of fit were investigated with the calibration plot and the Hosmer-Lemeshow test (HL). For these purposes the dataset was partitioned in 7 bins with an equal number of TL.

**References**

1.	Bunea F, She Y, Ombao H, Gongvatana A, Devlin K, Cohen R. Penalized least squares regression methods and applications to neuroimaging. *NeuroImage*. 2011;55(4):1519-1527. doi:10.1016/j.neuroimage.2010.12.028

2.	Zou H, Hastie T. Regularization and Variable Selection Via the Elastic Net. *Journal of the Royal Statistical Society Series B: Statistical Methodology*. 2005;67(2):301-320. doi:10.1111/j.1467-9868.2005.00503.x

3.	James G, Witten D, Hastie T, Tibshirani R. *An Introduction to Statistical Learning: With Applications in R.* Springer; 2013.
